# War exposure, post-traumatic stress symptoms and hair cortisol concentrations in Syrian refugee children

**DOI:** 10.1038/s41380-022-01859-2

**Published:** 2022-11-16

**Authors:** Demelza Smeeth, Fiona S. McEwen, Cassandra M. Popham, Elie G. Karam, John Fayyad, Dahlia Saab, Michael J. Rieder, Abdelbaset A. Elzagallaai, Stan van Uum, Michael Pluess

**Affiliations:** 1 Department of Biological and Experimental Psychology, School of Biological and Behavioural Sciences, Queen Mary University of London, London, UK; 2 Department of Psychiatry and Clinical Psychology, Balamand University, St George Hospital University Medical Center, Institute for Development, Research, Advocacy and Applied Care (IDRAAC), Beirut, Lebanon; 3 Department of Physiology and Pharmacology, Schulich School of Medicine and Dentistry, University of Western Ontario, London, ON Canada; 4 Division of Endocrinology and Metabolism, Schulich School of Medicine and Dentistry, University of Western Ontario, London, ON Canada

**Keywords:** Psychology, Biomarkers

## Abstract

Altered secretion of cortisol, the primary effector of the hypothalamus–pituitary–adrenal axis, has been proposed as a means by which traumatic experiences compromise later mental health. However, despite the popularity of cortisol as a potential biomarker for stress and adversity, findings are inconsistent, and little is known about the impact of war-related trauma on stress physiology of children and adolescents. Here we aimed to evaluate the relationships between war exposure, current living conditions, hair cortisol concentrations (HCC) and post-traumatic stress disorder (PTSD) symptoms in a large cohort of Syrian refugee children and adolescents (6–18 years) and their caregiver. This longitudinal observational study assessed Syrian refugee children and adolescents in two waves, 1 year apart, within informal tented settlements in Lebanon. The relationships between war exposure, time since leaving Syria, PTSD symptoms and HCC were investigated using linear mixed-model regression utilising both waves of data collected (Y1: *N* = 1574, Y2: *N* = 923). Hair cortisol concentration was positively, but weakly associated with the number of war-related events experienced. This was limited to those who were at least 12 years old at the time of war exposure. Conversely, HCC decreased with time since leaving Syria. HCC was also associated with PTSD symptoms but not with the quality of their current living conditions. This study revealed that changes to hypothalamic-pituitary-adrenal axis activity may accompany both earlier war exposure and current PTSD symptoms in children and adolescents. Additionally, early adolescence may be a particularly sensitive time in terms of trauma-related changes to the hypothalamic-pituitary-adrenal axis.

## Introduction

Exposure to psychologically stressful or traumatic life events is associated with the deterioration of mental health. This is especially true for those whose exposure occurs during childhood and adolescence which can increase risk of depression, externalising behaviours and post-traumatic stress disorder (PTSD) [1–4]. War is often accompanied by adverse life events including direct and indirect exposure to conflict, violence and bombardment, as well as subsequent forced displacement and factors relating to unsettled living circumstances [5]. These are associated with later poor mental health, particularly PTSD [3, 6–8]. During 2022, the number of displaced individuals globally rose above 100 million, many of whom had experienced war and were under the age of 18 [9, 10]. Therefore, a better understanding of the link between previous war exposure, current living conditions, and mental health is needed.

Studies have aimed to identify lasting biological signatures of adversity such as war exposure and displacement, and whether these mediate the risk of poor mental health. Cortisol is the primary effector of the hypothalamic–pituitary–adrenal (HPA) axis in physiological and psychological stress responses and its secretion is frequently altered in psychopathological states [11–14]. However, whether cortisol has utility as an biomarker of psychological adversity and poor mental health is less clear given differing findings across contexts, with reports of no association, hypersecretion and hyposecretion of cortisol [15, 16]. Results are also difficult to reconcile due to methodological differences. For example, cortisol can be measured in multiple tissues, but hair is easily accessible, particularly in humanitarian settings and in populations less amenable to invasive sampling, such as children. This method negates the impact of temporal hormone fluctuations and represents an assessment of averaged hormone secretion over recent months [17]. This measure of average HPA axis activity has shown that adversity is frequently associated with altered hair hormone concentration (HCC) levels in adults, but this is dependent on the type and timing of adversity as well as the current psychopathological state of the study population [15, 16]. Interestingly, reported psychologically traumatic experiences are often associated with elevated HCC in the short-term [18, 19], but more distant or repeated reported exposures correlate with hyposecretion in the long-term [13, 20].

Refugees are a unique population with experience of complex patterns of adversity, including potentially traumatic war-related events and often stressful adverse current living conditions. Several studies have found that cortisol secretion measured from hair is correlated with war exposure. For example, the number of war-related events experienced was positively associated with HCC in cohort studies comprising London-based adults, Ugandan adults and Libyan women [19, 21, 22]. Other studies have examined the impact of war and concurrent displacement on cortisol. In recently resettled refugees in Serbia, self-reported home and journey trauma (including war) was positively associated with fingernail cortisol, another cumulative cortisol measure, but this was not significant after controlling for covariates [23]. A further study aimed to disentangle the independent effects of displacement and ongoing insecurity [24]. Syrian refugee women settled in Germany exhibited lower HCC than long-settled Kurdish immigrant controls. Conversely, internally displaced Iraqi women living in refugee camps had a similar mean HCC to controls although variation was particularly high in this group.

Some evidence suggests that the specific type of war-related event has a differential relationship with cortisol. Studies utilising time-dependent measures of cortisol such as saliva have indicated that cortisol levels differ depending on the threatening nature of experienced events [25], the presence of sexual assault [26] or whether sustained injuries were disabling [27]. The timing of such events is also an important consideration given it has been argued that HCC is representative of more recent stressors [16]. However, findings in this regard are mixed. Time since immigration was positively associated with HCC in long-settled Kurdish asylum seekers and immigrants in Germany [24], whereas no association was found between time since settling or journey time and fingernail cortisol in recent refugees in Serbia [23].

Importantly, few studies have investigated the impact of refugee status or war exposure on HCC in children or adolescents. This is crucial, as these ages cover key stages in the development of the HPA-axis and studies have reported developmental-stage-specific impacts on the HPA axis following adversity [28]. Two cohort studies conducted on Palestinian adolescents within the Israeli-Palestinian conflict found no significant difference in HCC measures between those that reported traumatic war-related events and unexposed controls [29, 30]. Similarly, neither lifetime trauma exposure nor poverty was associated with HCC in Syrian refugee adolescents living in Jordan [31, 32]. However, the presence of PTSD symptoms following exposure to war was associated with elevated cross-sectional or longitudinal HCC measures [29, 31]. Few studies have assessed the relationship between PTSD and HCC in children, although lower HCC was found in children and adolescents with PTSD over 2 years following psychological trauma [33]. Conversely lifetime traumatic events were negatively associated with HCC in male unaccompanied refugee minors in Germany, whether considering the total number or number of different types of traumatic events [34]. These studies suggest that while war-related PTSD symptoms may be associated with elevated HCC in adolescents, the relationship between war exposure and HCC alone is unclear, particularly when compared to adults. These studies are also relatively small with samples ranging from 92 to 817 individuals (mean = 403) and have not explored this relationship within children. Furthermore, we are missing studies concerning those still living in adverse environments such as refugee camps.

Here we aim to explore the relationships between war exposure, the quality of current living conditions, HCC and PTSD in 1591 Syrian refugee children and adolescents. Firstly, we aim to examine the relationship between exposure to war-related events and HCC and whether this is dependent on the timing and specific type of war event. Secondly, we aim to examine the relationship between the quality of the current living conditions and HCC. Finally, we aim to identify relationships between PTSD symptoms and HCC, and whether HCC mediates the relationship between environmental exposures and PTSD symptoms. The large, longitudinal cohort used here features repeated measures and measurement of a wide range of important covariates. This is an understudied and highly representative population being minors and refugees who have not resettled in high-income countries and likely to still be experiencing significant stress related to their current living conditions. Compared to previous studies, this cohort also covers a broader age range allowing a better understanding of the effect of age at the time of exposure.

## Methods

### Sample

Participants came from a cohort of 1591 Syrian refugee child-caregiver dyads recruited from informal tented settlements in the Beqaa region of Lebanon for the Biological Pathways of Risk and Resilience in Syrian Refugee Children (BIOPATH) study [35]. An accelerated longitudinal design was used to collect data from multiple developmental stages over a short period of time given the high mobility of refugee populations. Child-caregiver dyads were eligible if they had left Syria within the previous four years, the child was aged 8–16 years at enrolment, and a primary caregiver was available. This age range was selected to examine children within a wide range of developmental stages while still ensuring that they were at a sufficient stage of development for questionnaire comprehension. Due to uncertainty about birth dates, some children outside the intended age range were recruited, and were retained within the sample unless there were concerns about comprehension.

Data was collected across two waves, 1 year apart from late 2017 to early 2019 with 1000 dyads successfully followed up (Supplementary Table 1). Follow-up was not possible if families could not be re-contacted, had moved outside of the study area or did not wish to re-participate. Followed-up participants were similar in many characteristics to dropouts, although, dropouts were more likely to be male, were slightly more advanced in pubertal development and tended to have left Syria more recently. However, similar war exposure, current living condition scores, PTSD symptoms and HCC were observed across groups.

Ethical approval was granted by the Institutional Review Board of the University of Balamand/Saint George Hospital University Medical Center, Lebanon (ref: IRB/O/024-16/1815), and the study was reviewed by the Lebanese National Consultative Committee on Ethics and approved by the Ministry of Public Health. Caregivers provided written informed consent after receiving a complete description of the study, while children provided assent. For more information see the BIOPATH cohort profile paper [35].

### Measures

#### Basic sociodemographic data

All participants were interviewed in their homes by Arabic-speaking interviewers at both data collection waves. Age, sex, nationality, and smoking status were reported on. The time since leaving Syria was reported as a categorical variable of 12-month time-intervals. Age at the end of war exposure was calculated by subtracting the time since leaving Syria time-interval midpoint from age. Pubertal development, body mass index, recent illness and any endocrinological illness or medication were recorded (see Supplementary Methods for further information).

#### War exposure, perceived refugee environment and PTSD symptoms

War exposure was measured using the War Events Questionnaire (WEQ), a 25-item checklist of war-related events [36]. Both child and caregiver reported on child-experienced events at Year 1 and each event was scored if reported by either interviewee, as previously recommended [37]. The main war exposure score utilised all questionnaire items and quantified the number of different war-related events experienced. Additional scores quantified war-related events which were particularly threatening to the child’s life or those around them. Additionally, children were categorised into six groups representing the most severe and proximal type of war-related event experienced (for more information see Supplementary Methods).

The perceived refugee environment index (PREI) was developed for the BIOPATH study as a multidimensional measure to assess the quality of the current living conditions of refugees. There is currently no comparable measure of the refugee environment, despite common features of these environments being linked to poor mental health in refugee children [38]. This caregiver-reported measure includes subscales assessing the quality of livelihood, basic needs, housing, access to services, family environment, community environment, working situation, future mobility, and learning environment with each item rated on a scale of 1–5 with higher scores indicated higher quality. For the purposes of this study, the total score was utilised, excluding questions relating to future mobility as these do not currently directly impact the child (e.g., “Do you expect that you will move to a different area in this country within the next 6 months?”). The sub-scales for livelihood, basic needs and housing were also utilised due to their direct impact on the child and previous reports of inadequate housing, poverty and malnutrition being associated with poor child mental health [39–41].

Child-reported PTSD symptoms were assessed using the Child PTSD Symptom Scale (CPSS), with minor changes made to phrasing and Arabic dialect following piloting. This scale had good validity in similar populations and showed acceptable reliability and validity against DSM-5 diagnosis of PTSD assessed via clinical interview in a representative subsample of the BIOPATH cohort [42].

### Hair cortisol

Hair sections at least 3 mm in diameter were cut from the posterior vertex, adjacent to the scalp using clean scissors, packaged in aluminium foil with the scalp end labelled and stored in a cool place. This totalled 1584 samples from year 1 and 924 samples from year 2. Participants also reported on frequency of hair washing and any hair alterations (e.g., hair dye, henna, or chemical straightening). Month of collection was noted to account for potential seasonal effects.

Cortisol concentration from hair segments (0–2 cm long) were assayed according to an established ELISA-based protocol and described fully in supplementary methods [19, 43]. HCC was normalised to sample weight and expressed as ng/g. For all analyses, HCCs were logged (base-10) to ensure normality. Ten year 1 samples and one year 2 sample were excluded due to outlying values of more than three standard deviations above the mean of the logged HCCs, resulting in a final sample of 1574 at year 1 and 923 at year 2.

### Statistical analysis

All analyses were conducted using R Studio (v.1.4.17) running R (v. 4.1.1). Initial associations of HCC with demographic, technical, and health-related characteristics were investigated using Mann–Whitney *U* test, Kruskal–Wallis *H* test and correlations for binary, categorical and continuous variables, respectively.

For main analyses, linear mixed models were fitted enabling the use of all waves of data collection available for each participant. This avoided the exclusion of potentially more at-risk individuals that couldn’t be reached at year 2. Analyses were run using the lme4 package [44], including HCC as the dependent variable and fixed effects of war exposure, current living conditions, time since leaving Syria or PTSD symptoms. We included analysis batch as a fixed effect and participant as a random intercept effect in all models. Minimal adequate models were constructed by including all potential covariates associated with HCC and reducing the model until only those which significantly contributed to the model remained. Covariates included age, sex, pubertal stage, nationality, smoking status, hair washing frequency, hair alterations and collection month.

Mediation analysis was conducted to test both HCC and PTSD symptoms as mediators, using the mediation R package [45]. Linear regression models were constructed with batch and sex as covariates using the year 1 data. We calculated direct and indirect effects and performed 5000 bootstrapped repetitions to calculate 95% confidence intervals and *p* values.

Sensitivity analyses were performed to ensure validity of results. Analyses were repeated excluding hair samples with unusual characteristics (e.g., coloured extracts), samples shorter than 2 cm, samples under the usual weight (20 mg), and children who reported any endocrinological illness or medication. Analyses were also repeated in the subset with biometric information controlling for standardised BMI *z*-scores.

## Results

### Sample characteristics

After removing outliers, HCCs remained for 1574 children at year 1 and 923 children at year 2 of data collection (Table 1 and Supplementary Table 2). Participants were aged 6–19 years old and evenly split between males and females (Y1: 47.4% male). HCC exhibited moderate within-individual correlation across timepoints (*r*(916) = 0.42, *p* < 0.001).

**Table 1:** Cohort demographics and relationship between demographics and HCC.

	Y1 (*N* = 1574)					Y2 (*N* = 923)				
Variable	*N*	%	Mean	SD	Test statistics	*N*	%	Mean	SD	Test statistics
Demographic										
Male^a^	746	47.4			*W* = 216,593, *p* < 0.001	396	42.9			*W* = 71,657, *p* < 0.001
Age			11.4	2.4	*r*(1572) = 0.34, *p* < 0.001			12.2	2.4	*r*(921) = 0.31, *p* < 0.001
Pubertal stage^b^					*W* = 98,781, *p* < 0.001					*W* = 41,976, *p* < 0.001
Pre-mid puberty	1300	82.6				705	76.4			
Late-post puberty	272	17.3				218	23.6			
Nationality					*Χ*^2^(3) = 10.63, *p* = 0.014					*Χ*^2^(3) = 2.62, *p* = 0.454
Syrian	1552	98.6				911	98.7			
Lebanese	8	0.5				3	0.3			
Palestinian	12	0.8				8	0.9			
Iranian	1	98.6				1	98.7			
Other	1	0.1				0	0			
Time since leaving Syria					*Χ*^2^(4) = 21.54, *p* < 0.001					*Χ*^2^(5) = 18.58, *p* = 0.002
0–12 months	290	18.4				4	0.4			
12–24 months	226	14.4				146	15.8			
24–36 months	218	13.9				118	12.8			
36–48 months	590	37.5				167	18.1			
48+ months	244	15.5				488	52.9			
Caregiver										
Mother	1406	89.3				839	90.9			
Father	64	4.1				22	2.4			
Stepmother	24	1.5				16	1.7			
Grandmother	24	1.5				14	1.5			
Sibling	27	1.7				6	0.7			
Other relative	25	1.6				9	1.0			
Non-relative	2	0.1				0	0			
Health-related		89.3								
BMI			18	3.6	*r*(901) = 0.27, *p* < 0.001			18.5	3.5	*r*(901) = 0.27, *p* < 0.001
Age and sex-adjusted BMI			−0.4	1.2	*r*(900) = 0.067, *p* = 0.043			−0.3	1.2	*r*(897) = 0.14, *p* < 0.001
Reported smoker^c^	20	1.3			*W* = 22,846, *p* < 0.001	17	1.8			*W* = 9295, *p* = 0.143
Recent illness^d^	762	48.4			*W* = 293,866.5, *p* = 0.086	514	55.7			*W* = 98,636, *p* = 0.107
Endocrinological illness or medication^e^	11	0.7			*W* = 7339, *p* = 0.403	7	0.8			*W* = 2205, *p* = 0.154
Hair-related										
Hair washing frequency					*Χ*^2^(2) = 2.85, *p* = 0.240					*Χ*^2^(2) = 1.10, *p* = 0.576
1–2 times/week	122	7.7				70	7.6			
3–4 times/week	962	61.1				524	56.8			
5+ times/week	489	31.1				356	38.6			
Hair alterations^f^					*W* = 216,593, *p* < 0.001	232	25.1			*W* = 90,960, *p* = 0.002
Hair colour					*Χ*^2^(2) = 7.72, *p* = 0.021					*Χ*^2^(2) = 0.64, *p* = 0.728
Black	1045	66.4				521	56.4			
Brown	510	32.4				349	37.8			
Other	19	1.2				53	5.7			
Psychological	*N*	%	Median	IQR		*N*	%	Median	IQR	
War exposure			9	5–14				9	5–14	
Most severe war event										
None	48	3				27	2.9			
Bombardment	105	6.7				63	6.8			
Other-directed violence	129	8.2				74	8			
Violence towards a close person	182	11.6				113	12.2			
Violence in the home	637	40.5				376	40.7			
Bodily harm	473	30.1				270	29.2			
Quality of current living conditions			3.2	2.9–3.6				3.3	3.0–3.7	
PTSD score			13.8	6–24				5	0–18	
Hair cortisol ng/g			73.6	32–175				86	46–181	

Reference groups for statistical tests: ^a^male, ^b^pre-mid puberty, ^c^No smoking, ^d^No illness, ^e^no illness or medication, ^f^no hair alterations. Missing data: 673 missing for Y1 BMI, 19 missing for Y2. 2 missing for Y1 pubertal stage. 6 missing for Y1 time since leaving Syria. 1 missing for Y1 smoking. 1 missing for Y1 hair washing frequency and 1 missing for Y2. 1 missing for Y1 hair alterations and 1 missing for Y2.

### War exposure, current living conditions and HCC

We first investigated whether the number of war-related events reported was associated with HCC utilising samples collected at both waves of data collection. Individuals reported anywhere from 0–24 of the 24 different war-related events asked about. The number of war-related events showed a small but positive association with HCC when controlling for only analysis batch, but controlling for age at interview, sex, batch, time since leaving Syria, pubertal stage, smoking, and hair colour led to a weaker and non-significant main effect of war-related events (Table 2). Age at interview was found to be the main confounder as its exclusion from the model revealed a significant fixed effect of war exposure equivalent to a 1.39% increase in HCC for each additional war-related event experienced. This equates to a 39.3% increase in HCC between those with no reported war-related events to those with the maximum number of war-related events reported. In comparison, the same model estimates females to have 62.7% higher HCC on average.

**Table 2:** Fixed effects of number of war-related events on hair cortisol concentration from linear mixed models.

Model	Fixed effect	*B*	SE	*p* value	% Change cortisol
Batch only	War exposure	0.006	0.002	0.002	1.3
Full model	War exposure	0.002	0.002	0.232	0.4
	Time: 12–24 months ago	−0.056	0.028	0.042	−12.2
	Time: 24–36 months ago	0.007	0.030	0.804	1.7
	Time: 36–48 months ago	−0.053	0.027	0.046	−11.5
	Time: 48+ months ago	−0.072	0.030	0.016	−15.3
	Sex	0.239	0.018	<0.001	73.4
	Age	0.053	0.004	<0.001	13.0
	Pubertal stage	0.080	0.025	0.001	20.4
	Smoking	−0.135	0.065	0.038	−26.7
	Hair colour: brown	−0.038	0.017	0.026	−8.4
	Hair colour: other	−0.083	0.046	0.070	−17.5
Full model excluding age	War exposure	0.004	0.002	0.010	1.0
	Time: 12–24 months ago	−0.059	0.028	0.039	−12.7
	Time: 24–36 months ago	0.008	0.031	0.799	1.8
	Time: 36–48 months ago	−0.060	0.027	0.029	−12.8
	Time: 48+ months ago	−0.087	0.031	0.005	−18.1
	Sex	0.211	0.019	<0.001	62.7
	Pubertal stage	0.251	0.021	<0.001	78.0
	Smoking	−0.237	0.066	<0.001	−42.1
	Hair colour: brown	−0.053	0.017	0.003	−11.4
	Hair colour: other	−0.078	0.047	0.098	−16.5

Linear mixed model specification: log10cortisol ~ war + batch + (1|participant) + additional covariates. Covariates in minimal model: age, sex, pubertal stage, smoking, hair colour. Fixed effects for analysis batch not shown due to the number of levels [46]. Reference groups: Sex (male), pubertal stage (early-mid puberty), hair colour (black). % change in cortisol calculated from regression *B* values. War exposure score range = 0–24.

Looking at the types of war-related event reported, we found that those that reported violence directed towards a close person or personal bodily harm as their most severe experience, exhibited elevated HCC, but this effect disappeared when controlling for covariates (Supplementary Table 3). Follow-up analyses based on these findings used scores which captured only the most potentially traumatising war-related events, i.e., those which potentially endangered their lives or people around them (Supplementary Table 4). While the number of personally life-threatening events was not significantly associated with HCC, the number of all observed life-threatening events was, and each additional event was associated with up to a 2.3% increase in HCC. However, results mirrored those when using the complete war exposure score and age at interview confounded this relationship.

Current living conditions of refugees are often characterised by a range of significant stressors including inadequate housing, nutrition, and poverty. We examined whether this current source of stress also contributed to altered HCC. Individuals scored anywhere from 1.6–4.9 in terms of the quality of their current living conditions on a scale from 1 (low quality) to 5 (high quality). We found that current living conditions were not associated with HCC whether looking at individual characteristics of the environment (e.g., basic needs, housing, or livelihood) or a cumulative score (Table 3 and Supplementary Table 5). These findings did not change when also controlling for war exposure (Supplementary Table 6).

**Table 3:** Fixed effects of the quality of current living conditions on hair cortisol concentration from linear mixed models.

Model	Fixed effect	*B*	SE	*p* value	% Change cortisol
Batch only	Quality of current living conditions	−0.005	0.016	0.743	−1.2
Full model	Quality of current living conditions	0.017	0.016	0.284	3.9
	Time: 12–24 months ago	−0.063	0.028	0.024	−13.5
	Time: 24–36 months ago	<0.001	0.030	1.000	0.0
	Time: 36–48 months ago	−0.066	0.027	0.014	−14.0
	Time: 48+ months ago	−0.085	0.030	0.005	−17.8
	Sex	0.239	0.018	<0.001	73.3
	Age	0.054	0.004	<0.001	13.2
	Pubertal stage	0.081	0.025	0.001	20.5
	Smoking	−0.141	0.065	0.030	−27.7
	Hair colour: brown	−0.038	0.017	0.026	−8.4
	Hair colour: other	−0.078	0.047	0.094	−16.4

Linear mixed model specification: log10cortisol ~ PREI + batch + (1|participant) + additional covariates. Covariates in minimal model: age, sex, pubertal stage, smoking, hair colour. Fixed effects for analysis batch not shown due to the number of levels [46]. Reference groups: Sex (male), pubertal stage (early-mid puberty), hair colour (black). % change in cortisol calculated from regression *B* values. PREI range = 0–4.89.

### Investigating time since war exposure

Next, we investigated whether time since the end of war exposure was associated with HCC using time since leaving Syria as a proxy, again using both waves of data collection (Table 2). Compared to those that left Syria within the previous 12 months, children who left more than 12 months ago (excluding 24–36 months ago) exhibited lower HCC even when controlling for the number of war-related events experienced. This is equivalent to a decrease in HCC of 12.1%, 11.5% and 15.2% for those who left Syria 12–24 months, 36–48 months and greater than 48 months ago, respectively.

We followed up on this relationship to investigate whether the relationship between the number of war-related events experienced and HCC differs when stratified by the time since leaving Syria (Supplementary Fig. 1). Despite the observed drop in HCC with time since the end of war exposure, there were no apparent differences in relationship between the number of war-related events and HCC between children whose exposure ended at different times.

### PTSD symptoms and HCC

We next aimed to identify associations between PTSD and HCC at both waves of data collection. Individuals scored anywhere from 0–51 on the PTSD symptom scale, covering the full range of the scale. PTSD symptoms were associated with a small increase in HCC when covarying for age, sex, batch, pubertal stage, and hair colour (Table 4). This represents a 0.32% increase in HCC for each point on the PTSD symptom scale, equivalent to a 17.7% increase in HCC between those who scored the minimum and maximum on the PTSD symptoms scale. In comparison, the same model estimates females to have 72.5% higher HCC than males.

**Table 4:** Fixed effects of post-traumatic stress disorder symptoms on hair cortisol concentration from linear mixed models.

Model	Fixed effect	*B*	SE	*p* value	% Change cortisol
Batch only	PTSD	0.003	0.001	<0.001	0.6
Full model	PTSD	0.001	0.001	0.041	0.3
	Sex	0.237	0.018	<0.001	72.5
	Age	0.056	0.004	<0.001	13.7
	Pubertal stage	0.070	0.025	0.005	17.6
	Hair colour: brown	−0.039	0.017	0.020	−8.6
	Hair colour: other	−0.076	0.046	0.100	−16.1
Full model excluding age	PTSD	0.002	0.001	0.003	0.4
	Sex	0.203	0.019	<0.001	59.7
	Pubertal stage	0.252	0.021	<0.001	78.5
	Hair colour: brown	−0.055	0.018	0.002	−11.9
	Hair colour: other	−0.075	0.047	0.116	−15.9

Linear mixed model specification: log_10_cortisol ~ ptsd + batch + (1|participant) + additional covariates. Covariates in minimal model: age, sex, pubertal stage, hair colour. Fixed effects for analysis batch not shown due to the number of levels [46]. Reference groups: Sex (male), pubertal stage (early-mid puberty), hair colour (black). % change in cortisol calculated from regression *B* values. PTSD symptom score range = 0–51.

### Investigating age at end of war exposure

**Fig. 1:**
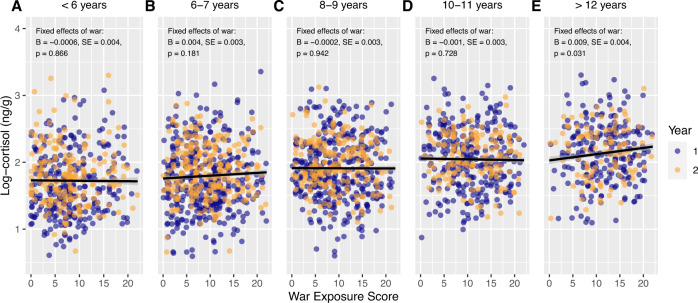
War exposure score plotted against logged hair cortisol concentration stratified by age at time of leaving Syria. Year 1 and year 2 samples are plotted for each individual where available (purple = Year 1, orange = Year 2). Lines represent the predicted population-level hair cortisol concentration for the number of war-related events on from linear mixed models controlling for sex, analysis batch and age at interview. Grey regions represent confidence intervals for the predicted values. The association between war exposure and cortisol is plotted separately for different age groups at the time of leaving Syria with **A** = younger than 6 years, **B** = 6-7 years, **C** = 8-9 years, **D** = 10-11 years, and **E** = older than 12 years.

Age at interview confounded the relationship between HCC and the number of war-related events, being associated with both HCC (*r* = 0.34, *p* < 0.001) and war exposure (*r* = 0.15, *p* < 0.001). We hypothesised that age at the time of war exposure may play a role in this relationship. Models were constructed for five groups stratified by age at cessation of exposure, controlling for batch, age at interview and sex (Fig. 1). Age groups were constructed to allow for approximately equal sized groups of narrow age ranges. While most age groups displayed null associations between war exposure and HCC, the oldest group (aged at least 12 years at the end of war exposure), showed a stronger relationship, particularly when compared to the entire cohort. This is equivalent to a 2.2% increase in HCC for each additional reported war-related event.

Given the similarity in observed relationship with PTSD symptoms and war exposure to HCC, similar age-stratified analyses were conducted between PTSD symptoms and HCC, and war exposure and PTSD symptoms (Supplementary Table 7). While all age groups exhibited an association between the number of war-related events and PTSD symptoms, this was stronger within the group that were at least 12 years at the end of war exposure. Conversely, there was no significant relationship between PTSD symptoms and HCC within any individual age group.

### HCC and PTSD symptoms as mediators

**Fig. 2:**
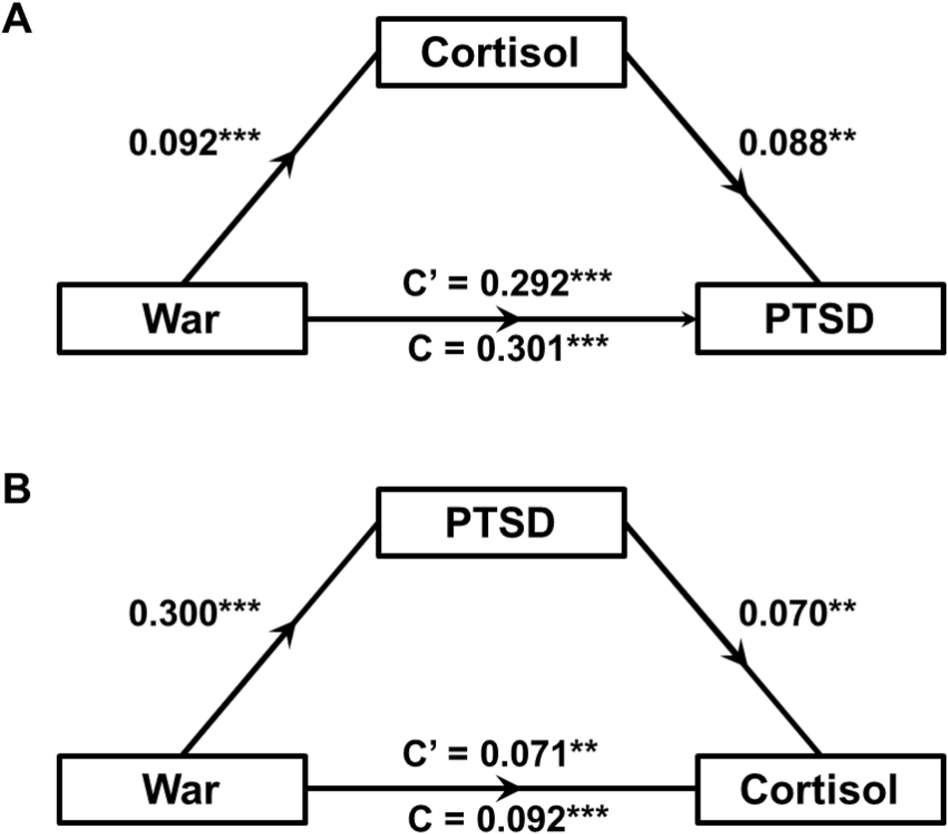
Pathway diagram for mediation models evaluating the relationships between war exposure, hair cortisol concentration (HCC) and post-traumatic stress disorder (PTSD) symptoms. **A** The model testing HCC as a mediator of the relationship between war exposure and PTSD symptoms. **B** The model testing PTSD symptoms as a mediator of the relationship between war exposure and HCC. Coefficients are standardised regression betas. C: non-mediated direct path, C’: mediated direct path. All paths: *p* < 0.01.

Finally, we investigated the potential mediating relationships between war exposure, hair cortisol and PTSD (Fig. 2). We did not examine the potential mediation effect with current living conditions due to the lack of observed relationship with HCC. These analyses were conducted using only the year 1 cross-sectional data due to the higher burden of PTSD symptoms within this wave as well as the closer proximity to war. We found evidence for partial mediation for both proposed models: the effect of war exposure on HCC was partially mediated via PTSD symptoms (ACME = 0.002, 95% CI [0.0007–0.0031], 22.9%) and the effect of war exposure on PTSD symptoms was also partially, but to a lesser degree, mediated via HCC (ACME = 0.018, 95% CI [0.006–0.033], 2.7%).

## Discussion

Within this large cohort of Syrian refugee children and adolescents we aimed to investigate the relationships between HCC, war exposure, current living conditions and PTSD symptoms. We found that HCC was positively associated with the number of war-related events after controlling for covariates (except age). However, effects were comparatively small with sex having approximately double the impact on HCC as experiencing the full range of war-related events. These results generally mirror previous findings in adults that exposure to war is associated with elevated HCC [19, 21, 22], but differ from the generally null findings in children [30, 31, 46]. Many existing studies found no association between war exposure and HCC, unless PTSD symptoms were also present. However, many of these studies were relatively small and possibly underpowered to detect the small effect sizes found here. The most similar population to the one studied here did not find an association between war exposure and HCC in a sample of Syrian refugee and Jordanian non-refugee youth [31]. However, they were living within urban centres rather than informal tented settlements possibly reflecting differences in ongoing stressors [47, 48]. Our findings are also in contrast with the previous finding that lifetime traumatic events, including war, were associated with lowered HCC in refugee youth in Germany [34]. However, together these may align with reports that hypersecretion of cortisol follows traumatic events if still experiencing insecure living conditions but then relative hyposecretion once this has subsided [18]. Other explanations may include differences in relative timing of war exposure or cohort characteristics such as age or sex.

Despite this relationship between the number of war-related events and HCC, we found no clear evidence of an impact of the type of war-related event experienced. There was an indication that more life-threatening events may have a stronger relationship with HCC. However, considering that more threatening war-related events tended to be accompanied by less threatening events, we cannot discount a cumulative effect of all war-related events. Our findings also support previous reports that time since exposure is inversely related with HCC [13, 49], indicating that while experiencing war could have lasting effects on the HPA axis, this may reduce over time and return to baseline. This lends support to the idea that HCC is associated with more recent stressors [50]. Alternatively, this decrease with time could be indicative of exposure-associated hyposecretion of cortisol [13, 20, 51]. However, without a sufficiently large no-exposure group this cannot be ascertained here.

We found that age at the time of interview confounded the relationship between war exposure and HCC with older children having higher HCC as well as greater war exposure. An elevated burden of war-related events in older children has been previously reported in numerous research contexts [52–54]. Older children may be more likely to spend a greater proportion of time outside their home and therefore are more likely to experience war-related events or may be more aware of the less proximal events surrounding them. Age-dependent memory formation and recall may also play a role [55]. However, our measure of war exposure utilises both child and caregiver reports potentially reducing the interference of age-associated recall in the child.

Further investigation of the age at the end of exposure found that the relationship between war exposure and HCC was limited to children that were 12 years or older at the time, even when controlling for age at time at interview. While this does not conclusively indicate that this subset of adolescents was driving the findings within the entire cohort, it does suggest that this age group was particularly affected by war exposure, supported by the stronger relationship between exposure and PTSD symptoms. Previous studies have found that adolescents exhibit heightened HPA-axis responses to adverse experiences as well as diminished effectiveness of stress buffering by caregivers [56–58]. This in turn can lead to heightened risk for developing trauma-related mental health problems following exposure in adolescence [59, 60]. Adolescence is an important time for the development of the HPA axis and accompanied by vast changes in steroid hormones [61]. Studies have also indicated that this period is accompanied by increased basal and stress-induced HPA axis activity and greater cortisol secretion [62] in addition to age-specific grey matter developmental changes in response to stress, including the anatomical components of the HPA axis [63, 64]. Younger children, on the other hand, may be somewhat shielded from the emotional impact of war-related events by their family, or may lack the cognitive skills to process the fear-associated qualities of war. For example, it has been shown that younger children tend to be less frightened by threats depicted on the news, while still being able to report on them accurately [65]. Therefore, the older group may have had heightened biological sensitivity to war exposure and a more lasting signature of adversity manifested in long-term elevated HCC or they may simply be more susceptible to such experiences. However, caution is recommended as this was an unplanned follow-up analysis and will need to be replicated in an independent cohort.

We found no evidence for a relationship between the quality of current living conditions and HCC even though living as a refugee in informal settlements has been associated with a wide range of potentially stressful experiences including limited access to basic needs, uncertain living circumstances and a lack of healthcare [66–68]. It is unclear why a relationship between current living conditions and HCC has not been observed. However, it should be noted that our measure was caregiver-reported and reported experiences may not be equally applicable to or perceived by the children of the family, particularly those who are younger.

We also found that children with greater PTSD symptom severity exhibit elevated HCC in line with previous studies in adults including those conducted with Syrian refugees [24] and Ugandan war-exposed individuals [22]. Fewer studies have investigated this relationship in minors, but there is some evidence for a positive association in younger war-affected populations [29, 31]. However, these effects are small with sex having approximately four times the impact on HCC as the maximum PTSD symptom burden. The mediation analyses indicated that both PTSD symptoms and HCC could feasibly mediate a small proportion of the relationship between war exposure and the outcome. However, it is unclear from these cross-sectional results whether the observed hypersecretion of cortisol leads to increased PTSD symptom burden, is a result of it, or whether both are independent outcomes of war exposure.

Despite the important findings, this study has some significant limitations. Firstly, war-related events were recalled retrospectively. While this study aimed to obtain the most accurate measure of these events through the questioning of both child and caregiver, we cannot rule out the inherent limitations with recall of such events, particularly in children. We were also limited in our measure of war-related events to the number of different types. Therefore, we are lacking information on repetition or chronicity of such events. Secondly, while we can be sure that war-related events occurred before the measurement of HCC, we cannot be certain whether war exposure has a direct impact on long-term HPA-axis changes or whether it is mediated by more immediate factors within their environment [5]. This is particularly important as it has been argued that HCC is representative of more recent stressors [16]. While we found no evidence that current living conditions were associated with HCC, other mediators may exist such as parent mental health, caregiving or physical activity [46, 69]. In addition experiencing war-related trauma has the potential to increase awareness of ongoing stressors in the immediate environment, as observed in Syrian refugee and Jordanian non-refugee children [70]. Furthermore, our measure of current stressors is parent-reported and may not capture the lived experiences of the child. Thirdly, the study lacked a true unexposed group for both war exposure and the current living conditions. While our cohort included a small number of children that reported zero war-related events (*n* = 48), all individuals were forcibly displaced and experienced the potential stressors of living in informal settlements. We measured and considered the quality of the current living conditions, but we acknowledge that even those reporting a higher quality environment most likely experience significant stressors. Future work will need to investigate how more recent experiences alter trauma-related cortisol secretion. Finally, despite finding that elevated HCC is weakly associated with both war exposure and PTSD symptom severity, caution is recommended given that key demographic variables such as age and sex were more strongly associated. This limits the utility as a predictive biomarker and highlights the importance of including these key variables within such analyses. In addition while we attempted to control for the majority of confounders, we may be missing unmeasured confounders such as physical activity [71].

## Conclusion

In conclusion, our findings found that long-term alterations to the HPA-axis, visible as elevated HCC, are associated with experience of war-related events and current PTSD symptoms in a large population of Syrian refugee children and adolescents. Firstly, the findings suggest that experiencing war is associated with a lasting up-regulation of the HPA axis visible as elevated HCC, but this may dissipate over time. This is especially true for children in the early stages of adolescence at the time of exposure, supporting the idea that adolescence is a time of relative sensitivity to adversity. However, it is unclear whether this is a direct effect or mediated by the more recent environment. Furthermore, elevated HCC was also indicative of greater burden of PTSD symptoms. However, effects were small, and questions remain regarding how long these disruptions to the HPA-axis remain, if disruptions remain visible as hypersecretion in the long-term, and whether they contribute to mental health in later life.

## Supplementary information

Supplementary Material
