# Supplementary material for: War exposure, post-traumatic stress symptoms and hair cortisol concentrations in Syrian refugee children
Source: Mol Psychiatry. 2022 Nov 16;28(2):647–56. doi: 10.1038/s41380-022-01859-2 (PMC9908541; doi:10.1038/s41380-022-01859-2)
Supplement: Supplementary file 1 — Supplementary Material [file 41380_2022_1859_MOESM1_ESM.docx]

**Supplementary Online Content for** ***War Exposure, Post-Traumatic Stress Symptoms and Hair Cortisol Concentrations in Syrian Refugee Children***

**Supplementary Methods**

- Demographic and health variables
- War exposure measures
- Linear mixed model analysis
- References

**Supplementary Tables**

- **Supplementary Table 1.** Cohort demographics at year 1 for those which were followed up at year 2 or dropped out.
- **Supplementary Table 2.** Technical variable summary and relationships between demographics and hair cortisol concentration
- **Supplementary Table 3.** Fixed effects of war event type on hair cortisol concentration from linear mixed models.
- **Supplementary Table 4.** Fixed effects of number of life-threatening war-related events on hair cortisol concentration from linear mixed models.
- **Supplementary Table 5.** Fixed effects of current living condition subscales on hair cortisol concentration from linear mixed models
- **Supplementary Table 6.** Fixed effects of number of war-related events and current living conditions on hair cortisol concentration from linear mixed models
- **Supplementary Table 7.** Fixed effects of PTSD symptoms on hair cortisol concentration and war exposure on PTSD symptoms stratified by age at time of leaving Syria

**Supplementary Figures**

- **Supplementary Figure 1.** War exposure score plotted against logged hair cortisol concentration stratified by time since leaving Syria.

## Supplementary methods

### Demographic variables

Pubertal development was assessed using a shortened version of the Puberty Development Scale ^1,2^, which limited questioning to initiation and age of menarche in girls, and facial hair growth and voice deepening in boys. Information on the general health of the children was reported on by both the child and caregiver, including acute and chronic physical health, injuries, dental problems, and medications, and when these were last experienced. For this study, any child- or parent-reported illness or injury that occurred during the time represented by the hair sample (previous 2 months) was flagged for removal in sensitivity analyses. In addition, any endocrinological illness or medication that could potentially impact hair cortisol concentration was also flagged for removal in sensitivity analyses. This predominantly comprised children with reported thyroid problems and those using topical corticosteroid medications. Biometric data, including height and weight were measured, starting part way through the first wave of data collection. Therefore, BMI measurements were unavailable for the first 680 children. Body mass index (BMI) was calculated in the standard manner. As BMI is highly dependent on sex and age in children and adolescents, BMI standard deviation scores (BMI SDS) were calculated using the childsds R package (v. 0.7.6) ^3,4^, with a Turkish reference population ^5^.

### War exposure measures

Both child and caregiver reported on child-experienced war events using the War Events Questionnaire (WEQ) and each event was scored if reported by either interviewee. WEQ item agreement ranged from 64.2% to 96.0% depending on item. In addition to the total number of war events experienced, two additional scores were created quantifying war events which were potentially threatening to the child’s life or people around them. Personally life-threatening events included kidnap, torture, injury, home invasion and personally experiencing bombardment in the home. Life threatening events which they personally experienced also included personally witnessing the kidnapping, killing, torture or injury of others.

To account for the varied nature of the war exposure, children were also classified into six groups representing the most severe and proximal type of war event experienced as described previously ^6^. Children were assigned to theoretical groupings based upon the type of war-related events reported: 1) no exposure, 2) indirect exposure to bombardment, 3) other-directed violence (i.e., towards strangers or acquaintances), 4) violence to a close person, 5) war-related violence in the home, and 6) personal harm. Most children fitted multiple categories but were assigned the most severe or proximal group category ^7^. For example, if a child reported indirect bombardment and personal harm, they were assigned to the personal harm group.

### Hair cortisol analysis

Cortisol concentration from hair segments (0–2 cm long) were assayed using an ELISA-based protocol. Hair sections were weighed, washed with isopropanol, and minced. Cortisol was extracted with methanol overnight at ambient temperature with shaking and evaporated under a stream of nitrogen gas at 60° C until dry. Resulting residues were reconstituted in phosphate-buffered saline before undergoing analysis in duplicate using a modified, commercially available immunoassay (11-CORHU-E01-SLV; ALPCO Diagnostics, USA). Hair color and unusual characteristics such as colored extract or sticky texture were noted. It was also noted if the sample weighed less than the target 20mg, if it was shorter than the target 2cm, or if the scalp end of the hair was unclear. Samples exceeding the ELISA’s reported detection range were diluted and reanalysed. Samples below the immunoassay detection limit (1 ng/mL) were not excluded from main analyses due to their bias towards younger females (Y1: *n* = 227, Y2: *n* = 66). The inter-assay coefficients of variation across plates were 6.5-9.8%. HCC was normalized to sample weight and expressed as ng/g. For all analyses, HCCs were logged (base-10) to ensure normality. Ten year 1 samples and one year 2 sample were excluded due to outlying values of more than three standard deviations above the mean of the logged HCCs, resulting in a final sample of 1574 at year 1 and 923 at year 2.

### Linear mixed model analysis

For the main analyses, we fitted linear mixed models using the lme4 package ^8^, including HCC as the dependent variable and added fixed effects of either war events, PTSD symptoms or current living conditions. We included analysis batch as a fixed effect and participant as a random intercept effect in all models. Covariates were included as additional fixed effects to check the potential impact of confounders. The model specification was as follows: log_10_cortisol ~ war OR ptsd OR current living conditions + batch + (1|participant) + additional covariates as main effects. Minimal adequate models were constructed by including all variables cross-sectionally associated with HCC at either timepoint and reducing the model until only those which significantly contributed to the model remained, in addition to our predictor of interest. Covariates included age, sex, pubertal stage, nationality, smoking status, hair washing frequency, hair alterations and collection month. BMI SDS was not included within models due to considerable non-random missingness within the first wave of data collection. P-values for the fixed effects of PTSD or war events on HCC were calculated using the lmerTest package, which applies Satterthwaite’s method to estimate degrees of freedom and generate p-values for mixed models ^9^. As all models used log-cortisol as an outcome, we calculated predicted percentage change in HCC from the model beta coefficients using the formula: % change = (10^Beta^-1)*100. Models were checked for the assumptions of linear models by inspecting scatter plots for non-linear relationships, residual plots for homoscedasticity and q-q plots for normality of residuals.

### Code and data availability

R code and data for all analyses is available upon request.

### References

1 Carskadon MA, Acebo C. A self-administered rating scale for pubertal development. *J Adolesc Health* 1993; **14**: 190–195.

2 Petersen AC, Crockett L, Richards M, Boxer A. A self-report measure of pubertal status: Reliability, validity, and initial norms. *J Youth Adolesc* 1988; **17**: 117–133.

3 Vogel M. *childsds: Data and Methods Around Reference Values in Pediatrics*. 2020https://CRAN.R-project.org/package=childsds (accessed 23 Jun2021).

4 Vogel M, Kirsten T, Kratzsch J, Engel C, Kiess W. A combined approach to generate laboratory reference intervals using unbalanced longitudinal data. *J Pediatr Endocrinol Metab* 2017; **30**: 767–773.

5 Bundak R, Furman A, Gunoz H, Darendeliler F, Bas F, Neyzi O. Body mass index references for Turkish children. *Acta Paediatr Oslo Nor 1992* 2006; **95**: 194–198.

6 Popham CM, McEwen FS, Karam E, Fayyad J, Karam G, Saab D *et al.* The dynamic nature of refugee children’s resilience: a cohort study of Syrian refugees in Lebanon. *Epidemiol Psychiatr Sci* 2022; **31**. doi:10.1017/S2045796022000191.

7 Regev S, Slonim-Nevo V. Trauma and mental health in Darfuri asylum seekers: The effect of trauma type and the mediating role of interpersonal sensitivity. *J Affect Disord* 2019; **246**: 201–208.

8 Bates D, Mächler M, Bolker B, Walker S. Fitting Linear Mixed-Effects Models Using lme4. *J Stat Softw* 2015; **67**: 1–48.

9 Kuznetsova A, Brockhoff PB, Christensen RHB. lmerTest Package: Tests in Linear Mixed Effects Models. *J Stat Softw* 2017; **82**: 1–26.

## Supplementary tables

| Supplementary Table 1. Cohort demographics at year 1 for those which were followed up at year 2 or dropped out. | | | | | | | | |
| --- | --- | --- | --- | --- | --- | --- | --- | --- |
|  | ***Followed-up (N = 918)*** | | | | ***Dropped out (N = 656)*** | | | |
| **Variable** | ***N*** | ***%*** | ***Mean*** | ***SD*** | ***N*** | ***%*** | ***Mean*** | ***SD*** |
| *Demographic* |  |  |  |  |  |  |  |  |
| Male | 394 | 42.9 |  |  | 352 | 53.7 |  |  |
| Age |  |  | 11.2 | 2.3 |  |  | 11.6 | 2.5 |
| Pubertal stage |  |  |  |  |  |  |  |  |
| Pre-mid puberty | 772 | 84.1 |  |  | 528 | 80.5 |  |  |
| Late-post puberty | 145 | 15.8 |  |  | 127 | 19.4 |  |  |
| Nationality |  |  |  |  |  |  |  |  |
| Syrian | 907 | 98.8 |  |  | 645 | 98.3 |  |  |
| Lebanese | 3 | 0.3 |  |  | 5 | 0.8 |  |  |
| Palestinian | 7 | 0.8 |  |  | 5 | 0.8 |  |  |
| Iranian | 1 | 98.8 |  |  | 0 | 98.3 |  |  |
| Other | 0 | 0 |  |  | 1 | 0.2 |  |  |
| Time since leaving Syria |  | | | | | | | |
| 0-12 months | 138 | 15 |  |  | 152 | 23.2 |  |  |
| 12-24 months | 123 | 13.4 |  |  | 103 | 15.7 |  |  |
| 24-36 months | 131 | 14.3 |  |  | 87 | 13.3 |  |  |
| 36-48 months | 309 | 33.7 |  |  | 281 | 42.8 |  |  |
| 48+ months | 217 | 23.6 |  |  | 27 | 4.1 |  |  |
| *Health-related* |  |  |  |  |  |  |  |  |
| BMI |  |  | 17.7 | 3.2 |  |  | 18.3 | 4 |
| Age & Sex-Adjusted BMI |  |  | -0.4 | 1.1 |  |  | -0.3 | 1.3 |
| Reported smoker | 9 | 1 |  |  | 11 | 1.7 |  |  |
| Recent illness | 463 | 50.4 |  |  | 299 | 45.6 |  |  |
| Endocrinological illness or medication | 7 | 0.8 |  |  | 4 | 0.6 |  |  |
| *Hair-related* |  |  |  |  |  |  |  |  |
| Hair washing frequency |  |  |  |  |  |  |  |  |
| 1-2 times/week | 70 | 7.6 |  |  | 52 | 7.9 |  |  |
| 3-4 times/week | 556 | 60.6 |  |  | 406 | 61.9 |  |  |
| 5+ times/week | 291 | 31.7 |  |  | 198 | 30.2 |  |  |
| Hair alterations | 263 | 28.6 |  |  | 162 | 24.7 |  |  |
| Hair color |  |  |  |  |  |  |  |  |
| Black | 603 | 65.7 |  |  | 442 | 67.4 |  |  |
| Brown | 305 | 33.2 |  |  | 205 | 31.2 |  |  |
| Other | 10 | 1.1 |  |  | 9 | 1.4 |  |  |
| Collection month |  |  |  |  |  |  |  |  |
| September | 0 | 0 |  |  | 0 | 0 |  |  |
| October | 303 | 33 |  |  | 199 | 30.3 |  |  |
| November | 323 | 35.2 |  |  | 196 | 29.9 |  |  |
| December | 248 | 27 |  |  | 155 | 23.6 |  |  |
| January | 44 | 4.8 |  |  | 106 | 16.2 |  |  |
| *Technical* |  |  |  |  |  |  |  |  |
| Colored extract | 8 | 0.9 |  |  | 0 | 0 |  |  |
| Short samples | 110 | 12 |  |  | 83 | 12.7 |  |  |
| Unknown hair end | 106 | 11.5 |  |  | 77 | 11.7 |  |  |
|  |  |  |  |  |  |  |  |  |
| *Psychological* | ***N*** | ***%*** | ***Median*** | ***IQR*** | ***N*** | ***%*** | ***Median*** | ***IQR*** |
| War-related events |  |  | 9 | 5 to 14 |  |  | 9 | 5 to 14 |
| Most severe war event |  |  |  |  |  |  |  |  |
| None | 27 | 2.9 |  |  | 21 | 3.2 |  |  |
| Bombardment | 62 | 6.8 |  |  | 43 | 6.6 |  |  |
| Other-directed violence | 72 | 7.8 |  |  | 57 | 8.7 |  |  |
| Violence towards a close person | 112 | 12.2 |  |  | 70 | 10.7 |  |  |
| Violence in the home | 375 | 40.8 |  |  | 262 | 39.9 |  |  |
| Bodily harm | 270 | 29.4 |  |  | 203 | 30.9 |  |  |
| Quality of current living conditions |  |  | 3.2 | 2.9 to 3.6 |  |  | 3.2 | 2.8 to 3.5 |
| PTSD Score |  |  | 13 | 6 to 24 |  |  | 14 | 6 to 24 |
| Hair Cortisol ng/g |  |  | 75.4 | 32.8 to 181.2 |  |  | 71.0 | 31.6 to 166.1 |
| Missing data: 1 missing pubertal stage for followed-up, 1 missing pubertal stage for dropped out. 6 missing time since leaving Syria for dropped out. 1 missing smoking status for followed-up. 1 missing hair washing frequency for followed-up. 1 missing hair alterations for followed-up. | | | | | | | | |

| Supplementary Table 2. Technical variable summary and relationships between demographics and hair cortisol concentration | | | | | | |
| --- | --- | --- | --- | --- | --- | --- |
|  | ***Y1 (N = 1 574)*** | | | ***Y2 (N = 923)*** | | |
| ***Variable*** | ***N*** | ***%*** | ***Test statistics*** | ***N*** | ***%*** | ***Test statistics*** |
| Collection month |  |  | Χ^2^(3) = 198.3, p < 0.001 |  |  | Χ^2^(4) = 0.82, p = 0.936 |
| September | 0 | 0 |  | 36 | 3.9 |  |
| October | 502 | 31.9 |  | 334 | 36.2 |  |
| November | 519 | 33.0 |  | 260 | 28.2 |  |
| December | 403 | 25.6 |  | 172 | 18.6 |  |
| January | 150 | 9.5 |  | 121 | 13.1 |  |
| Extraction batch |  |  | Χ^2^(26) = 342.13, p < 0.001 |  |  | Χ^2^(19) = 51.09, p < 0.001 |
| Colored extract ^a^ | 8 | 0.5 | W = 7647.5, p = 0.281 | 28 | 3 | W = 13518, p = 0.477 |
| Short samples ^b^ | 193 | 12.3 | W = 152659.5, p = 0.001 | 14 | 1.5 | W = 6540, p = 0.858 |
| Unknown hair end ^c^ | 183 | 11.6 | W = 142090.5, p = 0.010 | 75 | 8.1 | W = 40712, p < 0.001 |
| Reference groups for statistical tests: ^a^ no coloured extract, ^b^ sufficient length, ^c^ known scalp end. | | | | | | |

| Supplementary Table 3. Fixed effects of war event type on hair cortisol concentration from linear mixed models | | | | | |
| --- | --- | --- | --- | --- | --- |
| ***Model*** | ***Fixed Effect*** | ***B*** | ***SE*** | ***p-value*** | ***% Change*** |
| Batch only | War event type: Bombardment | 0.077 | 0.067 | 0.255 | 19.4 |
|  | War event type: Other-directed violence | 0.081 | 0.066 | 0.214 | 20.6 |
|  | War event type: Violence towards a close person | 0.127 | 0.063 | 0.044 | 34.0 |
|  | War event type: Violence in the home | 0.078 | 0.058 | 0.181 | 19.6 |
|  | War event type: Bodily harm | 0.123 | 0.059 | 0.037 | 32.7 |
|  |  |  |  |  |  |
| Full model | War event type: Bombardment | 0.003 | 0.058 | 0.963 | 0.6 |
|  | War event type: Other-directed violence | -0.002 | 0.056 | 0.967 | -0.5 |
|  | War event type: Violence towards a close person | -0.014 | 0.054 | 0.790 | -3.3 |
|  | War event type: Violence in the home | -0.014 | 0.050 | 0.775 | -3.2 |
|  | War event type: Bodily harm | 0.010 | 0.051 | 0.848 | 2.3 |
|  | Time: 12-24 months ago | -0.057 | 0.028 | 0.042 | -12.2 |
|  | Time: 24-36 months ago | 0.004 | 0.030 | 0.882 | 1.0 |
|  | Time: 36-48 months ago | -0.058 | 0.026 | 0.027 | -12.5 |
|  | Time: 48+ months ago | -0.078 | 0.030 | 0.009 | -16.4 |
|  | Sex | 0.235 | 0.018 | < 0.001 | 71.6 |
|  | Age | 0.055 | 0.004 | < 0.001 | 13.4 |
|  | Pubertal stage | 0.079 | 0.025 | 0.002 | 20.0 |
|  | Hair colour: brown | -0.038 | 0.017 | 0.027 | -8.3 |
|  | Hair colour: other | -0.087 | 0.046 | 0.059 | -18.1 |
|  |  |  |  |  |  |
| Full model excluding age | War event type: Bombardment | 0.048 | 0.060 | 0.425 | 11.6 |
|  | War event type: Other-directed violence | 0.057 | 0.058 | 0.331 | 13.9 |
|  | War event type: Violence towards a close person | 0.052 | 0.056 | 0.355 | 12.7 |
|  | War event type: Violence in the home | 0.043 | 0.052 | 0.406 | 10.4 |
|  | War event type: Bodily harm | 0.073 | 0.052 | 0.166 | 18.2 |
|  | Time: 12-24 months ago | -0.060 | 0.029 | 0.034 | -13.0 |
|  | Time: 24-36 months ago | 0.001 | 0.031 | 0.982 | 0.2 |
|  | Time: 36-48 months ago | -0.070 | 0.027 | 0.010 | -14.9 |
|  | Time: 48+ months ago | -0.098 | 0.031 | 0.001 | -20.3 |
|  | Sex | 0.201 | 0.019 | < 0.001 | 58.8 |
|  | Pubertal stage | 0.259 | 0.021 | < 0.001 | 81.5 |
|  | Hair colour: brown | -0.052 | 0.018 | 0.003 | -11.4 |
|  | Hair colour: other | -0.083 | 0.047 | 0.081 | -17.4 |
| Linear mixed model specification: log10cortisol ~ war + batch + (1\|participant) + additional covariates. Fixed effects for analysis batch not shown due to the number of levels (46). Reference groups: Sex (male), pubertal stage (early-mid puberty), hair color (black). | | | | | |

| Supplementary Table 4. Fixed effects of number of life-threatening war-related events on hair cortisol concentration from linear mixed models | | | | | | | | | | | |
| --- | --- | --- | --- | --- | --- | --- | --- | --- | --- | --- | --- |
|  |  |  | ***Personally life-threatening events*** | | | |  | ***All life-threatening events*** | | | |
| ***Model*** | ***Fixed Effect*** | ***B*** | | ***SE*** | ***p-value*** | ***% Change*** | ***B*** | | ***SE*** | ***p-value*** | ***% Change*** |
| Batch only | War exposure | 0.024 | | 0.011 | 0.046 | 5.6 | 0.012 | | 0.004 | 0.001 | 2.9 |
|  |  |  | |  |  |  |  | |  |  |  |
| Full model | War exposure | 0.018 | | 0.010 | 0.068 | 4.2 | 0.006 | | 0.003 | 0.062 | 1.4 |
|  | Time: 12-24 months ago | -0.057 | | 0.028 | 0.040 | -12.3 | -0.056 | | 0.028 | 0.045 | -12.0 |
|  | Time: 24-36 months ago | 0.007 | | 0.030 | 0.815 | 1.6 | 0.009 | | 0.030 | 0.756 | 2.2 |
|  | Time: 36-48 months ago | -0.055 | | 0.026 | 0.038 | -11.8 | -0.051 | | 0.026 | 0.053 | -11.1 |
|  | Time: 48+ months ago | -0.073 | | 0.030 | 0.014 | -15.6 | -0.070 | | 0.030 | 0.019 | -15.0 |
|  | Sex | 0.239 | | 0.018 | < 0.001 | 73.6 | 0.239 | | 0.018 | < 0.001 | 73.4 |
|  | Age | 0.054 | | 0.004 | < 0.001 | 13.1 | 0.053 | | 0.004 | < 0.001 | 13.0 |
|  | Pubertal stage | 0.079 | | 0.025 | 0.002 | 19.9 | 0.081 | | 0.025 | 0.001 | 20.4 |
|  | Smoking | -0.132 | | 0.065 | 0.042 | -26.2 | -0.132 | | 0.065 | 0.042 | -26.2 |
|  | Hair color: brown | -0.037 | | 0.017 | 0.028 | -8.3 | -0.038 | | 0.017 | 0.026 | -8.4 |
|  | Hair color: other | -0.085 | | 0.046 | 0.066 | -17.7 | -0.082 | | 0.046 | 0.075 | -17.2 |
|  |  |  | |  |  |  |  | |  |  |  |
| Full model excluding age | War exposure | 0.019 | | 0.010 | 0.053 | 4.6 | 0.010 | | 0.003 | 0.003 | 2.3 |
|  | Time: 12-24 months ago | -0.061 | | 0.029 | 0.033 | -13.1 | -0.058 | | 0.028 | 0.041 | -12.5 |
|  | Time: 24-36 months ago | 0.003 | | 0.031 | 0.918 | 0.7 | 0.009 | | 0.031 | 0.773 | 2.1 |
|  | Time: 36-48 months ago | -0.067 | | 0.027 | 0.013 | -14.3 | -0.060 | | 0.027 | 0.029 | -12.8 |
|  | Time: 48+ months ago | -0.095 | | 0.031 | 0.002 | -19.7 | -0.087 | | 0.031 | 0.005 | -18.2 |
|  | Sex | 0.210 | | 0.019 | < 0.001 | 62.3 | 0.211 | | 0.019 | < 0.001 | 62.6 |
|  | Pubertal stage | 0.252 | | 0.021 | < 0.001 | 78.8 | 0.251 | | 0.021 | < 0.001 | 78.1 |
|  | Smoking | -0.242 | | 0.066 | < 0.001 | -42.7 | -0.236 | | 0.066 | < 0.001 | -41.9 |
|  | Hair color: brown | -0.053 | | 0.017 | 0.003 | -11.4 | -0.053 | | 0.017 | 0.003 | -11.4 |
|  | Hair color: other | -0.082 | | 0.047 | 0.082 | -17.2 | -0.077 | | 0.047 | 0.103 | -16.3 |
| Linear mixed model specification: log10cortisol ~ war + batch + (1\|participant) + additional covariates. Fixed effects for analysis batch not shown due to the number of levels (46). Reference groups: Sex (male), pubertal stage (early-mid puberty), hair color (black). For personally life-threatening events, war exposure score range = 0-5. For all life-threatening events, war exposure score range = 0-12. % change in cortisol calculated from regression B values. | | | | | | | | | | | |

| Supplementary Table 5. Fixed effects of current living condition subscales on hair cortisol concentration from linear mixed models | | | | | |
| --- | --- | --- | --- | --- | --- |
| ***Model*** | ***Fixed Effect*** | ***B*** | ***SE*** | ***p-value*** | ***% Change cortisol*** |
| Batch only | Basic needs | -0.002 | 0.010 | 0.858 | -0.4 |
|  | Housing | -0.005 | 0.011 | 0.667 | -1.1 |
|  | Livelihood | -0.007 | 0.008 | 0.408 | -1.5 |
|  |  |  |  |  |  |
| Full model | Basic needs | 0.004 | 0.009 | 0.638 | 1.0 |
|  | Housing | 0.003 | 0.011 | 0.769 | 0.7 |
|  | Livelihood | 0.001 | 0.007 | 0.872 | 0.3 |
|  |  |  |  |  |  |
| Full model excluding age | Basic needs | 0.002 | 0.010 | 0.867 | 0.4 |
|  | Housing | 0.005 | 0.011 | 0.633 | 1.2 |
|  | Livelihood | < 0.001 | 0.008 | 0.998 | 0.0 |
| Linear mixed model specification: log10cortisol ~ environment + batch + (1\|participant) + additional covariates. Covariates in minimal model: age, sex, pubertal stage, smoking, hair colour. Fixed effects for additional covariates not included for simplicity. Refugee environment subscale range = 0-5. % change in cortisol calculated from regression B values. | | | | | |

| Supplementary Table 6. Fixed effects of number of war-related events and current living conditions on hair cortisol concentration from linear mixed models | | | | | |
| --- | --- | --- | --- | --- | --- |
| ***Model*** | ***Fixed Effect*** | ***B*** | ***SE*** | ***p-value*** | ***% Change cortisol*** |
| Batch only | War exposure | 0.006 | 0.002 | 0.001 | 1.3 |
|  | Current living conditions | 0.002 | 0.017 | 0.910 | 0.4 |
|  |  |  |  |  |  |
| Full model | War exposure | 0.002 | 0.002 | 0.157 | 0.5 |
|  | Current living conditions | 0.019 | 0.016 | 0.216 | 4.6 |
|  | Time: 12-24 months ago | -0.062 | 0.028 | 0.026 | -13.4 |
|  | Time: 24-36 months ago | 0.004 | 0.030 | 0.905 | 0.8 |
|  | Time: 36-48 months ago | -0.061 | 0.027 | 0.025 | -13.1 |
|  | Time: 48+ months ago | -0.079 | 0.031 | 0.010 | -16.6 |
|  | Sex | 0.240 | 0.018 | < 0.001 | 73.8 |
|  | Age | 0.053 | 0.004 | < 0.001 | 13.1 |
|  | Pubertal stage | 0.081 | 0.025 | 0.001 | 20.6 |
|  | Smoking | -0.134 | 0.065 | 0.039 | -26.6 |
|  | Hair colour: brown | -0.037 | 0.017 | 0.031 | -8.1 |
|  | Hair colour: other | -0.075 | 0.047 | 0.107 | -15.9 |
|  |  |  |  |  |  |
| Full model excluding age | War exposure | 0.005 | 0.002 | 0.005 | 1.1 |
|  | Current living conditions | 0.022 | 0.016 | 0.171 | 5.2 |
|  | Time: 12-24 months ago | -0.065 | 0.029 | 0.023 | -13.9 |
|  | Time: 24-36 months ago | 0.003 | 0.031 | 0.915 | 0.8 |
|  | Time: 36-48 months ago | -0.068 | 0.028 | 0.014 | -14.6 |
|  | Time: 48+ months ago | -0.094 | 0.032 | 0.003 | -19.5 |
|  | Sex | 0.212 | 0.019 | < 0.001 | 63.0 |
|  | Pubertal stage | 0.251 | 0.021 | < 0.001 | 78.3 |
|  | Smoking | -0.238 | 0.066 | < 0.001 | -42.2 |
|  | Hair colour: brown | -0.052 | 0.018 | 0.003 | -11.3 |
|  | Hair colour: other | -0.073 | 0.048 | 0.126 | -15.5 |
| Linear mixed model specification: log10cortisol ~ war + environment + batch + (1\|participant) + additional covariates. Covariates in minimal model: age, sex, pubertal stage, smoking, hair colour. Fixed effects for analysis batch not shown due to the number of levels (46). Reference groups: Sex (male), pubertal stage (early-mid puberty), hair colour (black). % change in cortisol calculated from regression B values. War exposure score range = 0-24. Current living conditions range = | | | | | |

| Supplementary Table 7. Fixed effects of PTSD symptoms on hair cortisol concentration and war exposure on PTSD symptoms stratified by age at time of leaving Syria | | | | | |
| --- | --- | --- | --- | --- | --- |
| ***Model*** | ***Age at exposure*** | ***B*** | ***SE*** | ***p-value*** | ***% Change cortisol*** |
| HCC ~ PTSD symptoms | <6 years | 0.002 | 0.001 | 0.241 | 0.39 |
|  | 6-7 years | 0.001 | 0.001 | 0.287 | 0.30 |
|  | 8-9 years | 0.002 | 0.001 | 0.177 | 0.39 |
|  | 10-11 year | -0.0001 | 0.001 | 0.945 | -0.02 |
|  | 12+ years | 0.002 | 0.002 | 0.240 | 0.45 |
|  |  |  |  |  |  |
| PTSD symptoms ~ War exposure | <6 years | 0.458 | 0.106 | < 0.001 | - |
|  | 6-7 years | 0.508 | 0.090 | < 0.001 | - |
|  | 8-9 years | 0.555 | 0.098 | < 0.001 | - |
|  | 10-11 year | 0.653 | 0.106 | < 0.001 | - |
|  | 12+ years | 0.801 | 0.138 | < 0.001 | - |
| Linear mixed model specification: Model + batch + (1\|participant) + age at interview + sex. Fixed effects for additional covariates not included for simplicity. % change in cortisol calculated from regression B values where transformed HCC variables are used. | | | | | |

##
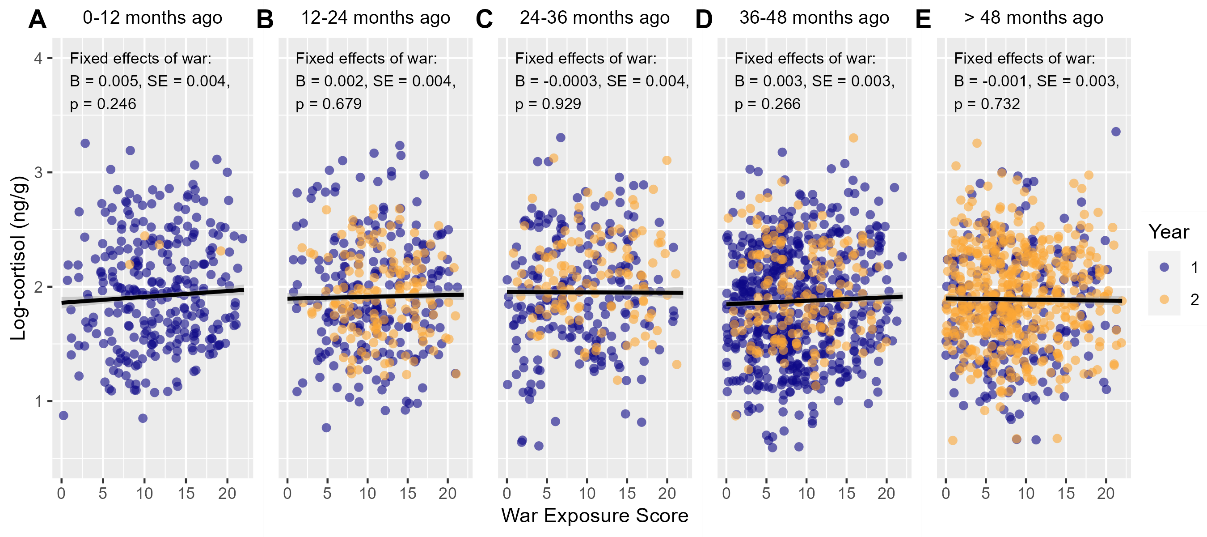
Supplementary figures

Supplementary Figure 1. War exposure score plotted against logged hair cortisol concentration stratified by time since leaving Syria. Year 1 and year 2 samples are plotted for each individual where available (Purple = Year 1, orange = Year 2). Lines represent the predicted population-level hair cortisol concentration for the number of war-related events on from linear mixed models controlling for sex, analysis batch and age at interview. Grey regions represent confidence intervals for the predicted values.
